# Age at first childbirth and the risk of hypertriglyceridemia among Korean women

**DOI:** 10.4178/epih.e2023010

**Published:** 2022-12-29

**Authors:** Hye Rin Choi, Hyeon Chang Kim

**Affiliations:** 1 Center for Cohort Studies, Total Healthcare Center, Kangbuk Samsung Hospital, Sungkyunkwan University School of Medicine, Seoul, Korea; 2 Institute of Medical Research, Sungkyunkwan University School of Medicine, Suwon, Korea; 3 Department of Preventive Medicine, Yonsei University College of Medicine, Seoul, Korea

**Keywords:** Childbirth, Parturition, Hypertriglyceridemia, Triglycerides

## Abstract

**OBJECTIVES:**

We aimed to investigate the association of age at first childbirth with the risk of hypertriglyceridemia among Korean women.

**METHODS:**

This study used data from the Korean Genome and Epidemiology Study–Cardiovascular Disease Association Study. In total, 16,747 women were included in the cross-sectional analysis, and 6,250 women were included in the longitudinal analysis. The participants were divided based on their age at first childbirth (<20, 20-24, 25-29, and ≥30 years). Hypertriglyceridemia was defined as triglyceride levels of ≥150 mg/dL.

**RESULTS:**

The multivariable-adjusted odds ratio for prevalent hypertriglyceridemia was 1.19 (95% confidence interval [CI], 1.01 to 1.40) in women whose first childbirth was before 20 years of age, compared to those whose first childbirth was at 25-29 years of age, after adjustment for age, study site, body mass index, blood pressure, diabetes, alcohol consumption, carbohydrate intake, income, marital status, education, parity, usage of oral contraceptives, and hormone replacement status. During a median follow-up of 5.2 years, 1,770 women developed hypertriglyceridemia. Compared with women who gave birth to their first child between 25 years and 29 years of age, those giving birth to their first child before 20 years of age had a higher risk for incident hypertriglyceridemia in later life (adjusted hazard ratio, 1.25; 95% CI, 0.99 to 1.57).

**CONCLUSIONS:**

Giving birth to one’s first child before the age of 20 years was associated with an increased risk of hypertriglyceridemia among Korean women.

## GRAPHICAL ABSTRACT

[Fig f3-epih-45-e2023010]

## INTRODUCTION

Pregnancy and childbirth are major life events affecting metabolic function and the cardiovascular system [1,2]. Blood lipid profiles constantly change during menstruation, pregnancy, childbirth, and menopause [3-5]. In particular, serum triglyceride levels have been reported to predict maternal or fetal adverse health effects [6-9]. Estrogen also regulates lipoprotein metabolism. Increased estrogen levels reduce lipoprotein lipase secretion, thus elevating blood triglyceride levels [10,11]. As estrogen could cause severe hypertriglyceridemia [12,13], some researchers have suggested that clinicians should check blood triglyceride levels in reproductive-age women before initiating estrogen therapy.

First childbirth before the age of 20 years has been linked to an increase in adverse cardiovascular health outcomes and metabolic disorders [14]. Pregnancy and childbirth in adolescence have been linked to a high risk of hypertriglyceridemia because changes in the lipid profile during pregnancy are consistent with the hormonal changes associated with the onset of puberty [15-17]. Most previous studies have investigated whether early menarche [18], early natural menopause [4,19], or abortions [20] were associated with metabolic syndrome [21]. However, these studies did not focus on the age at first childbirth. Furthermore, in the 1960s in Korea, women living in rural areas often got married early. Hence, they gave birth at an early age [22]. In this study, we targeted inhabitants of rural areas with a relatively diverse childbirth history to identify factors that might be associated with hypertriglyceridemia in later life. Therefore, this research evaluated whether the age at first childbirth was associated with adverse triglyceride levels in Korean women living in rural communities.

## MATERIALS AND METHODS

### Study population

This is a rural community-based cohort study that used data from the Korean Genome and Epidemiology Study–Cardiovascular Disease Association Study, which investigated the risk factors for cardiovascular disease (CVD) development [23]. Study participants aged ≥ 40 years at baseline were recruited between 2005 and 2011 from 11 rural communities in Korea. Follow-up examinations were conducted 4 times until 2016, after the baseline study. The mean follow-up duration was 5.2 years. In total, 28,337 participants (10,821 men and 17,516 women) completed the health examinations and questionnaires at baseline.

**Figure 1.**
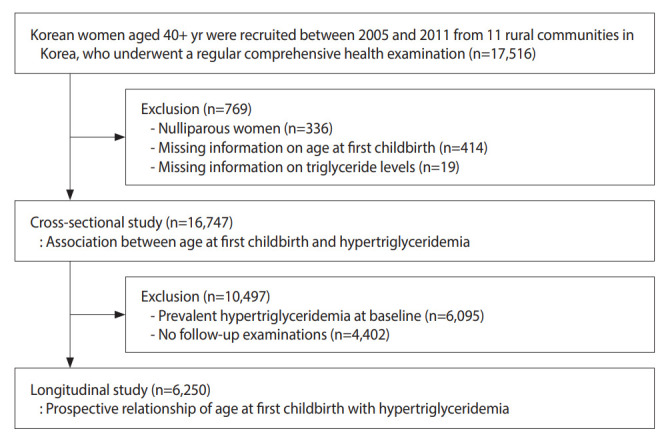
Flow diagram for the selection of the study population.

Nulliparous women (n= 336) and women not having information about age at first childbirth (n= 414) or triglyceride levels (n= 19) were excluded from this cohort study. Finally, 16,747 women were included for a cross-sectional analysis. We included only women without hypertriglyceridemia at baseline for the longitudinal analysis to determine whether childbirth factors could affect newer-onset hypertriglyceridemia. Women who participated in at least 1 follow-up examination were included. Therefore, a total of 6,250 women were included in the investigation on longitudinal associations (Figure 1).

### Measurements

Based on a predefined protocol, all participants were individually interviewed using standardized questionnaires by trained interviewers. The reproductive factors that were included were age at first childbirth, parity, age at menarche, menopausal status, usage of oral contraceptives, and hormone replacement status. The women were asked questions about their age at first childbirth, such as “Have you experienced pregnancy and delivery; if yes, then what was your age at first childbirth?”. Based on their age at first childbirth, we divided the study participants into 4 groups as follows: <20 years, 20-24 years, 25-29 years (reference), and ≥30 years. A history of health-related behaviors, including alcohol consumption, smoking status, and physical activity was taken. The participants were categorized as current drinkers or former/never drinkers based on their alcohol consumption. The participants were dichotomized by smoking status into current smokers or former/never smokers. However, the proportion of current smokers was very low; hence, we did not include smoking status in the analysis of this study. Physical activity was indicated by “yes” for participants who reported exercising enough to sweat at least once a week and “no” for those who did not. The socioeconomic status included household income, occupation, education, and marital status. Household income was defined as the average income of the household members per month and was divided into two groups: < 1,000 US dollar (USD) per month and ≥ 1,000 USD per month. The occupation was categorized as white, pink, or blue collar and others (housewives, students, and unemployed). Education levels were also classified into 2 levels: participants whose education was an elementary school level or lower and those who had an education level higher than elementary school. Marital status was dichotomized as living with a spouse or living alone (i.e., single, separated, divorced, and widowed). Participants wore lightweight clothing and no shoes for convenient and reliable examinations. The standing height was measured to the nearest 0.1 cm on a stadiometer, and body weight was measured to the nearest 0.1 kg on a digital scale. Body mass index (BMI) was calculated by dividing the body weight in kilograms by the height in meters squared (kg/m^2^). Blood pressure was recorded after participants rested for more than 5 minutes. Systolic and diastolic blood pressure were measured twice with at least a 5-minute interval between measurements using a standard mercury sphygmomanometer (Baumanometer; WA Baum, Copiague, NY, USA) and an automatic sphygmomanometer (Dinamap 1846 SX/P; GE Healthcare, Milwaukee, WI, USA) depending on the institution. Additional measurements were taken if the difference between the first and second measurements was greater than 10 mmHg. The average of the last 2 measurements was used for analysis. Hypertension was defined as a systolic blood pressure of ≥140 mmHg, a diastolic blood pressure of ≥ 90 mmHg, or the current use of blood pressure-lowering drugs.

After at least 8 hours of fasting, blood samples of all participants were collected from the antecubital vein. Total cholesterol, high-density lipoprotein cholesterol, and triglyceride levels were determined using an enzymatic method (ADVIA 1800; Siemens Healthineers, Deerfield, IL, USA). Fasting glucose levels were assayed by a colorimetric method (ADVIA 1800; Siemens Healthineers), and fasting insulin levels were measured with an immunoradiometric assay (SR-300; Stratec, Birkenfeld, Germany). Hypertriglyceridemia was defined as a triglyceride level ≥ 150 mg/dL. Diabetes mellitus was defined as fasting glucose level ≥ 126 mg/dL or current treatment with oral antidiabetic agents or insulin.

### Statistical analysis

Data were presented as mean values with standard deviations or numbers with percentages. For continuous variables with a skewed distribution, data were presented as median values with interquartile ranges. In order to investigate the linear trends of characteristics according to age at first childbirth, general linear models with contrast coefficients for linear trend tests for continuous variables were performed. The Cochran–Armitage test was also used for linear trends for categorical variables. Triglyceride values were log-transformed for parametric analysis because they were right-skewed. Multivariable logistic regression analysis was performed to examine the cross-sectional association between age at first childbirth and prevalent hypertriglyceridemia, using only baseline data. To adjust for potential confounders, model 1 was adjusted for age, study site, BMI, menopausal status (only for the total sample), blood pressure, and diabetes; model 2 was additionally adjusted for alcohol consumption, carbohydrate intake, income, marital status, and education; and model 3 was additionally adjusted for parity, usage of oral contraceptives and hormone replacement status. We selected the covariates from previous studies [14,24] and decided on the final adjusted model by considering the statistical implications. A sensitivity analysis was also conducted for women who had never taken lipid-lowering medication.

Additionally, Cox proportional hazard models were conducted to determine whether the age at first childbirth affected the risk of new-onset hypertriglyceridemia in the follow-up period in both the total sample and postmenopausal women. In this analysis, we excluded participants who already had baseline hypertriglyceridemia or those who did not participate in any follow-up examinations. We defined the index date as the first health examination date when an individual’s serum triglyceride level was over 150 mg/dL. Person-years of follow-up were calculated from the initial examination to either the time of occurrence of hypertriglyceridemia or the date of the last health check-up, whichever came first. Hazard ratios (HRs) and 95% confidence interval (CIs) for developing hypertriglyceridemia during the follow-up period were estimated after adjusting for age, study site, BMI, menopausal status (only for the total sample), blood pressure, diabetes, alcohol consumption, carbohydrate intake, income, marital status, education, parity, usage of oral contraceptive pills, and hormone replacement status. We considered potential confounders that were measured at baseline visits. The proportional hazards assumption was checked before using the Cox proportional hazard regression model. The cumulative incidence of hypertriglyceridemia was drawn using Kaplan–Meier curves from the time-to-event analysis. Furthermore, the log-rank test was performed to compare the time to incident hypertriglyceridemia among the groups defined by age at first childbirth.

All statistical analyses were conducted using SAS version 9.4 (SAS Institute Inc., Cary, NC, USA) and R version 3.6.2 (R Foundation for Statistical Computing, Vienna, Austria). Statistical significance was defined as a 2-sided p-value of less than 0.05, and marginal significance was defined as a 2-sided p-value of less than 0.10.

### Ethics statement

The study protocol was approved by the Institutional Review Board of Severance Hospital, Yonsei University College of Medicine (Y-2019-0170). All participants provided written informed consent.

## RESULTS

Table 1 describes the baseline characteristics according to the age at first childbirth. Among 16,747 participants, 1,176 (7.0%) had their first childbirth at < 20 years, 9,283 (55.4%) from 20 to 24 years, 5,441 (32.5%) from 25 to 29 years, and 847 (5.1%) at ≥ 30 years. As the age at first childbirth increased, the mean age at baseline decreased significantly and the proportions of women with ≥ 5 children and blue-collar workers decreased significantly. Furthermore, BMI, waist circumference, systolic and diastolic blood pressure, fasting glucose and insulin, total cholesterol, and triglyceride levels decreased significantly as the age at first childbirth increased. Younger age at first childbirth was associated with a lower proportion of women living with their spouses and higher proportions of women with lower education levels and income less than 1,000 USD per month.

Table 2 shows the cross-sectional associations between the age at first childbirth and prevalent hypertriglyceridemia at baseline among all participants and postmenopausal women. Overall, 41.6% of all women and 43.4% of postmenopausal women who had their first childbirth before the age of 20 had hypertriglyceridemia at the baseline examination. In the unadjusted model, the odds ratios (ORs) of prevalent hypertriglyceridemia were 1.78 (95% CI, 1.56 to 2.02) for women with their first childbirth at < 20 years and 1.35 (95% CI, 1.25 to 1.45) for women with their first childbirth between 20 years and 24 years, compared with those with their first birth at age 25-29. The negative associations between age at first childbirth and prevalent hypertriglyceridemia were significant (p < 0.001). After adjusting for age, study site, BMI, menopausal status (only for the total sample), blood pressure, diabetes, alcohol consumption, carbohydrate intake, income, marital status, education, parity, usage of oral contraceptives, and hormone replacement status, the association remained significant. Compared to women with their first childbirth between 25 years and 59 years old, the fully adjusted OR was 1.19 (95% CI, 1.00 to 1.40) for prevalent hypertriglyceridemia in the group with their first birth before age 20. When analyzed as a continuous variable, the OR of prevalent hypertriglyceridemia corresponding to a one-year decrease in the age of first childbirth was 1.01 (95% CI, 1.00 to 1.03). In the sensitivity analysis, we only included postmenopausal women. The results were similar to the main findings. The fully adjusted ORs for having hypertriglyceridemia at baseline were 1.19 (95% CI, 1.01 to 1.40) for women with their first childbirth at < 20 years and 1.06 (95% CI, 0.96 to 1.17) for those with their first childbirth at 20-24 years, compared to the group with their first childbirth at age 25-59. As shown in Supplementary Material 1, the age at first childbirth was inversely and significantly correlated with log-transformed triglyceride levels regardless of age adjustment. Furthermore, after excluding women who took lipidlowering medications, the associations between the age at first childbirth and prevalent hypertriglyceridemia remained significant (Supplementary Material 2). Women who experienced their first childbirth before 20 years had a higher OR (1.20; 95% CI, 1.01 to 1.42) for having hypertriglyceridemia than the reference group.

Table 3 shows the longitudinal associations between age at first childbirth and the incidence of hypertriglyceridemia at follow-up visits in all participants and postmenopausal women. Supplementary Material 3 shows a comparison of baseline characteristics between respondents and non-respondents to follow-up examinations. In all women, the fully adjusted hazard ratios (HRs) for incident hypertriglyceridemia were 1.25 (95% CI, 0.99 to 1.57) and 1.22 (95% CI, 1.06 to 1.40) for women who had their first childbirth at < 20 years and 20-24 years, respectively, compared with the group with their first birth at 25-29 years old. In a sensitivity analysis, postmenopausal women with their first childbirth at < 20 years and 20-24 years had significantly higher HRs (1.25; 95% CI, 0.99 to 1.57 and 1.22; 95% CI, 1.06 to 1.40) for developing hypertriglyceridemia during the follow-up period than those with their first childbirth between 25 years and 29 years, after adjusting for age at baseline, study site, BMI, blood pressure, diabetes, alcohol consumption, carbohydrate intake, socioeconomic status, parity, usage of oral contraceptives, and hormone replacement status. Furthermore, when we restricted the participants to those who underwent at least 3 follow-up health examinations as a sensitivity analysis, the adjusted HRs for incident hypertriglyceridemia were 1.69 (95% CI, 1.11 to 2.58) and 1.35 (95% CI, 1.05 to 1.74) in those with first childbirth at < 20 years and 20-24 years, respectively, compared to the reference group (Supplementary Material 4).

**Figure 2.**
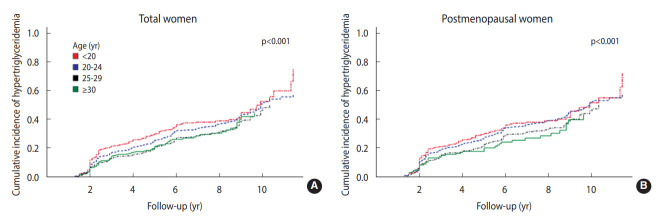
Cumulative incidence of hypertriglyceridemia according to age groups at first childbirth in the total sample (A) and postmenopausal women (B) using Kaplan-Meier plots.

The cumulative incidence of hypertriglyceridemia based on age at first childbirth using Kaplan–Meier plots in the total sample and postmenopausal women is displayed in Figure 2. In women with their first childbirth at < 20 years, the onset of hypertriglyceridemia was earlier than in women who were older at their first childbirth. The difference between the 2 groups of age at first childbirth was also significant (p < 0.001). Among postmenopausal women, new-onset hypertriglyceridemia occurred significantly earlier in those who were younger at their first childbirth (p<0.001). Women who had their first childbirth at ≥ 30 years of age were more likely to develop late-onset hypertriglyceridemia.

## DISCUSSION

This study examined whether an independent association of age at first childbirth with prevalent or incident hypertriglyceridemia was present among Korean women living in rural areas. We found that an early age at first childbirth (i.e., < 20 years) was significantly associated with a higher likelihood of prevalent hypertriglyceridemia at baseline compared to women whose first childbirth occurred between 25 years and 29 years, in both the total sample and postmenopausal women. During the follow-up period, women with their first childbirth at < 20 years had a 26% higher risk of incident hypertriglyceridemia than those with their first childbirth at 25-29 years. Among postmenopausal women, the risk of hypertriglyceridemia in later life increased by about 3% as the age of first childbirth decreased by 1 year. Furthermore, in both total and postmenopausal women, early first childbirth hastened the development of hypertriglyceridemia.

Our results were consistent with the results of previous studies [14,15,24-27]. A cross-sectional study of 4,262 postmenopausal women found that early pregnancy (maternal age at first delivery ≤ 20 years) was significantly associated with an increased risk of metabolic syndrome. Among the determinants of metabolic syndrome, the odds of having high triglyceride levels were 33.3% higher in women experiencing their first delivery at ≤ 20 years than in those experiencing their first delivery at ≥ 26 years [14]. A prospective cohort study in the United States with a 10-year follow-up suggested that adolescent pregnancy had atherogenic effects on blood lipids independent of weight gain after childbirth. Atherogenic lipid profiles from adolescence to adulthood are important risk factors for CVD events. Therefore, in women experiencing their first childbirth at ≤ 20 years, the lipid profile should be carefully monitored [15]. In a preliminary study using data from the International Mobility in Aging study, the authors found that adolescent childbirth was significantly associated with a high risk of CVD. As age at first childbirth increased, the Framingham risk scores decreased. Early age of first childbirth was associated with significantly higher Framingham risk scores for CVD than in nulliparous women [24]. A review suggested that an early age at first childbirth resulted in adverse physiological and sociological outcomes throughout the lifespan. In women with their first childbirth before 20 years of age, the risk of overall CVD events increased [25]. Both previous papers observed an association between age at first childbirth and CVD outcomes. Since the prevalence of hypertriglyceridemia is a major risk factor for CVD, those results could support our findings about the association between the age at first childbirth and maternal hypertriglyceridemia in later life. Furthermore, women with their first childbirth before 20 years of age had a higher likelihood of developing elevated triglyceride levels even postpartum because pregnant teenagers might be exposed to higher risks of adverse physical changes than adult pregnant women. Pregnancy-related changes contributed to longer exposure to CVD risk factors, including hypertriglyceridemia, than in women with their first childbirth in adulthood [24]. An earlier age at first birth was significantly associated with a higher BMI, body weight, and waist circumference [14]. Obesity induced by childbirth might have a significant relationship with hypertriglyceridemia in later life. Even though previous studies have investigated the biological mechanisms between adolescent childbirth and hypertriglyceridemia, the exact mechanism has not been identified.

The current study has several limitations. First, there might have been recall bias about participants’ reproductive history, which was collected by self-reported questionnaires. Moreover, women’s reproductive information might not have been accurate because visiting an obstetrics and gynecology clinic was less commonplace when many of the participants were pregnant. Second, as hypertriglyceridemia could be confirmed only based on an assessment of blood triglyceride levels, misclassification of hypertriglyceridemia cases could have taken place. Third, the diagnosis date of incident hypertriglyceridemia could not be identified. However, we found that young age at first childbirth might be associated with an increased risk of incident hypertriglyceridemia over time. Thus, the results of the longitudinal analysis provided additional evidence for the causal association of first childbirth with hypertriglyceridemia in later life. Fourth, although we considered several potential confounders, it is possible that unmeasured factors, including reproductive hormones (e.g., estrogen or follicle-stimulating hormone), could have affected the association of first childbirth with incident hypertriglyceridemia. As with the use of reproductive hormones, further longitudinal research could be conducted to assess whether changes in estrogen levels according to childbirth-related characteristics are associated with the development of hypertriglyceridemia. Lastly, this study was conducted among Korean women living in rural communities. Therefore, it is difficult to generalize the results to other ethnic populations.

This study found that giving birth to one’s first child before 20 years of age might lead to high triglyceride levels, resulting in cardiovascular health problems in later life. Reproductive history was found to be independently predictive of CVD and might be useful for CVD prevention efforts. In women whose first childbirth occurred before 20 years of age, targeted preventive actions should be developed to manage lipid profiles. Further studies are needed to investigate the underlying mechanisms.

## Figures and Tables

**Figure f3-epih-45-e2023010:**
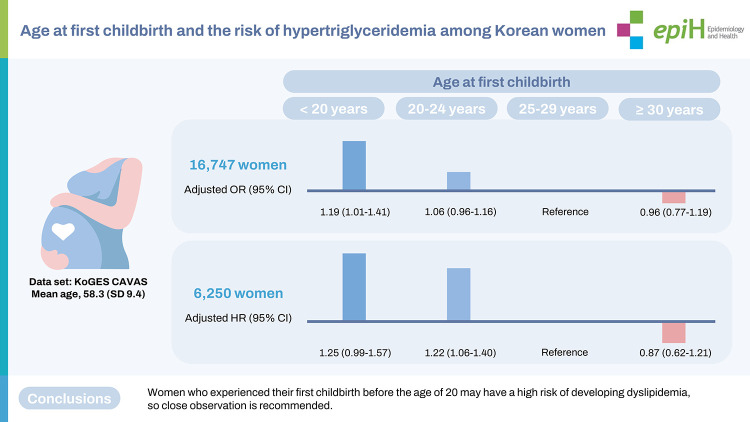


**Table 1.** Baseline characteristics according to age groups at first childbirth

Variables	Age at first childbirth (yr)				p for trend
	<20 (n=1,176)	20-24 (n=9,283)	25-29 (n=5,441)	≥30 (n=847)	
Age (yr)	64.2±9.9	59.7±9.0	54.8±8.5	53.3±8.7	<0.001
Age at first childbirth (yr)	18.5±0.7	22.1±1.4	26.4±1.3	32.4±2.9	<0.001
Parity					
1-2	104 (8.9)	1,979 (21.4)	2,761 (50.8)	679 (80.2)	<0.001
3-4	413 (35.1)	4,444 (48.0)	2,228 (41.0)	154 (18.2)	
≥5	658 (56.0)	2,844 (30.7)	449 (8.3)	14 (1.6)	
Menarche age (yr)	16.0± 2.4	16.9±2.5	16.8±3.1	16.6±4.0	<0.001
Oral contraceptives	356 (30.2)	2,941 (31.8)	1,238 (22.8)	122 (14.5)	<0.001
Hormone replacement status	84 (8.4)	870 (12.2)	655 (18.9)	85 (17.7)	<0.001
Postmenopausal status	1,053 (89.5)	7,666 (82.6)	3,705 (68.1)	507 (59.9)	<0.001
Living with spouse	686 (58.8)	7,012 (75.8)	4,626 (85.5)	728 (86.7)	<0.001
Low education level (≤elementary school)	1,103 (93.8)	7,239 (78.0)	2,514 (46.2)	263 (31.1)	<0.001
Low household income (<1,000 USD/mo)	948 (80.6)	6,310 (68.0)	2,356 (43.3)	290 (34.2)	<0.001
Occupation					
White collar	10 (0.9)	145 (1.6)	364 (6.7)	103 (12.2)	<0.001
Pink collar	82 (7.0)	871 (9.4)	812 (14.9)	113 (13.3)	
Blue collar	719 (61.1)	5,201 (56.0)	2,060 (37.9)	216 (25.5)	
Others	365 (31.0)	3,066 (33.0)	2,205 (40.5)	415 (49.0)	
Body mass index (kg/m²)	25.1±3.5	24.7±3.3	24.3±3.1	24.2±3.4	<0.001
Waist circumference (cm)	85.3±8.9	83.6±8.8	81.8±8.7	81.2±9.1	<0.001
Systolic BP (mmHg)	128.5±18.8	126.4±18.1	123.5±18.2	121.9±17.9	<0.001
Diastolic BP (mmHg)	79.4±11.3	78.8±11.0	77.6±11.3	76.9±11.4	<0.001
Fasting glucose (mg/dL)	95 [88, 104]	92 [86, 101]	91 [85, 98]	91 [85, 98]	<0.001
Fasting insulin (μIU/mL)	8.2 [6.7, 10.4]	7.7 [6.1, 9.9]	7.4 [5.9, 9.6]	7.4 [5.9, 9.6]	0.012
Total cholesterol (mg/dL)	205.6±38.7	205.4±37.5	201.5±36.6	200.2±37.4	<0.001
HDL cholesterol (mg/dL)	45.3±10.5	46.1±10.7	46.5±10.9	47.7±11.5	<0.001
Triglycerides (mg/dL)	134 [97, 187]	124 [89, 176]	111 [82, 160]	108 [76, 153]	<0.001
Hypertriglyceridemia	489 (41.6)	3,253 (35.0)	1,557 (28.6)	216 (25.5)	<0.001
Diabetes	192 (16.3)	1,093 (11.8)	491 (9.0)	71 (8.4)	<0.001
Hypertension	259 (22.0)	1,300 (14.0)	476 (8.8)	78 (9.2)	<0.001
Dyslipidemia	640 (54.4)	4,772 (51.4)	2,530 (46.5)	362 (42.7)	<0.001
Regular physical activity	255 (21.7)	2,521 (27.2)	2,091 (38.5)	343 (40.5)	<0.001
Current smokers	68 (5.8)	221 (2.4)	62 (1.1)	33 (3.9)	<0.001
Current drinkers	342 (29.1)	2,606 (28.1)	1,448 (26.6)	245 (28.9)	0.132
Energy intake (kcal/day)	1,432.5± 445.9	1,556±542.3	1,656±583.5	1,684.4±620.0	<0.001

Values are presented as mean± standard deviation, median [interquartile range], or number (%). USD, US dollar; BP, blood pressure; HDL, high-density lipoprotein.

**Table 2.** Cross-sectional associations of age at first childbirth with the prevalence of hypertriglyceridemia at baseline in the total sample and postmenopausal women

Age at first childbirth			No. of women	Case, n (%)^1^	OR (95% CI) for hypertriglyceridemia			
					Unadjusted	Model 1^2^	Model 2^3^	Model 3^4^
Total women								
	Continuous, per 1 yr earlier		16,747	5,515 (32.9)	1.06 (1.05, 1.07)	1.02 (1.01, 1.03)	1.01 (1.00, 1.02)	1.01 (1.00, 1.03)
	Category							
		<20	1,176	489 (41.6)	1.78 (1.56, 2.02)	1.17 (1.02, 1.35)	1.11 (0.97, 1.29)	1.19 (1.01, 1.40)
		20-24	9,283	3,253 (35.0)	1.35 (1.25, 1.45)	1.08 (0.99, 1.16)	1.03 (0.95, 1.11)	1.06 (0.96, 1.16)
		25-29	5,441	1,557 (28.6)	1.00 (reference)	1.00 (reference)	1.00 (reference)	1.00 (reference)
		≥30	847	216 (25.5)	0.85 (0.72, 1.01)	0.91 (0.76, 1.07)	0.95 (0.80, 1.13)	0.96 (0.77, 1.19)
	p for trend				<0.001	0.003	0.128	<0.001
Postmenopausal women								
	Continuous, per 1 yr earlier		12,931	4,667 (36.1)	1.05 (1.04, 1.06)	1.03 (1.01, 1.04)	1.02 (0.01, 1.03)	1.01 (1.00, 1.03)
	Category							
		<20	1,053	457 (43.4)	1.62 (1.41, 1.86)	1.27 (1.10, 1.48)	1.24 (1.07, 1.44)	1.19 (1.01, 1.40)
		20-24	7,666	2,867 (37.4)	1.26 (1.16, 1.37)	1.12 (1.03, 1.23)	1.09 (1.00, 1.20)	1.06 (0.96, 1.17)
		25-29	3,705	1,192 (32.2)	1.00 (reference)	1.00 (reference)	1.00 (reference)	1.00 (reference)
		≥30	507	151 (29.8)	0.89 (0.73, 1.10)	0.90 (0.73, 1.11)	0.93 (0.76, 1.15)	0.96 (0.77, 1.19)
	p for trend				<0.001	<0.001	0.002	<0.001

OR, odds ratio; CI, confidence interval.

1 Cases for hypertriglyceridemia.

2 Model 1: adjusted for age, study site, body mass index, menopausal status (only for the total sample), blood pressure, and diabetes.

3 Model 2: adjusted for age, study site, body mass index, menopausal status (only for the total sample), blood pressure, diabetes, alcohol consumption, carbohydrate intake, income, marital status, and education.

4 Model 3: adjusted for age, study site,, body mass index, menopausal status (only for the total sample), blood pressure, diabetes, alcohol consumption, carbohydrate intake, income, marital status, education, parity, usage of oral contraceptives, and hormone replacement status.

**Table 3.** Longitudinal associations between age at first childbirth at the baseline visit and incidence of hypertriglyceridemia at a follow-up visit

Age at first childbirth			No. at risk	Events^1^	PY	Incidence rate (/100 PY)	HR (95% CI) for hypertriglyceridemia		
							Unadjusted	Model 1^2^	Model 2^3^
Total women									
	Continuous, per 1 yr earlier		6,250	1,770	32,360	5.47	1.04 (1.02, 1.05)	1.02 (1.00, 1.03)	1.02 (1.00, 1.04)
	Category								
		<20	440	142	2,096.2	6.77	1.44 (1.20, 1.74)	1.22 (1.00, 1.49)	1.25 (0.99, 1.57)
		20-24	3,350	1,012	17,332.2	5.84	1.23 (1.11, 1.37)	1.15 (1.03, 1.29)	1.22 (1.06, 1.40)
		25-29	2,120	532	11,189.2	4.75	1.00 (reference)	1.00 (reference)	1.00 (reference)
		≥30	340	84	1,742.4	4.82	1.01 (0.81, 1.28)	1.05 (0.83, 1.32)	0.87 (0.62, 1.21)
	p for trend						<0.001	0.023	0.001
Postmenopausal women									
	Continuous, per 1 yr earlier		4,584	1,376	23,433.9	5.87	1.03 (1.02, 1.05)	1.02 (1.00, 1.04)	1.02 (1.00, 1.04)
	Category								
		<20	382	121	1,804.6	6.71	1.33 (1.08, 1.64)	1.17 (0.94, 1.46)	1.25 (0.99, 1.57)
		20-24	2,661	846	13,513.1	6.26	1.24 (1.09, 1.40)	1.18 (1.03, 1.35)	1.22 (1.06, 1.40)
		25-29	1,357	364	7,142	5.1	1.00 (reference)	1.00 (reference)	1.00 (reference)
		≥30	184	45	974.2	4.62	0.90 (0.66, 1.23)	0.90 (0.66, 1.22)	0.87 (0.62, 1.21)
	p for trend						<0.001	0.023	0.001

PY, person-years; HR, hazard ratio; CI, confidence interval.

1 Hypertriglyceridemia events.

2 Model 1: adjusted for age, study site, body mass index, menopausal status (only for the total sample), blood pressure, diabetes, alcohol consumption, carbohydrate intake, income, marital status, and education;

3 Model 2: adjusted for age, study site, body mass index, menopausal status (only for the total sample), blood pressure, diabetes, alcohol consumption, carbohydrate intake, income, marital status, education, parity, usage of oral contraceptives, and hormone replacement status.

## SUPPLEMENTARY MATERIALS

Supplementary Material 1. Correlation between age at first childbirth and log-transformed triglyceride levels with scatter plots in total (A) and postmenopausal (B) women

Supplementary Material 2 Cross-sectional association of age at first childbirth with prevalence of hypertriglyceridemia at baseline in total and postmenopausal women who had never used lipid-lowering medications

Supplementary Material 3 Comparison of baseline characteristics between respondents and non-respondents to follow-up examinations

Supplementary Material 4 Longitudinal association between age at first childbirth at the baseline visit and incidence of hypertriglyceridemia at a follow-up visit among participants who underwent at least three follow-up examinations
