# Supplementary material for: Age at first childbirth and the risk of hypertriglyceridemia among Korean women
Source: Epidemiol Health. 2022 Dec 29;45:e2023010. doi: 10.4178/epih.e2023010 (PMC10106550; doi:10.4178/epih.e2023010)
Supplement: Supplementary Material 1. — Correlation between age at first childbirth and log-transformed triglyceride levels with scatter plots in total (A) and postmenopausal (B) women [file epih-45-e2023010-Supplementary-Fig-1.docx]

| 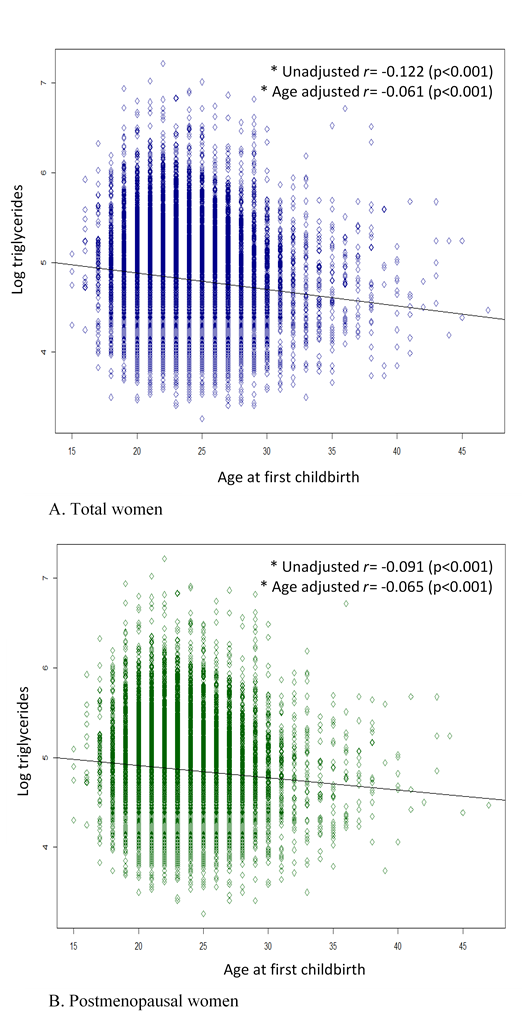 |
| --- |
| Supplementary Material 1. Correlation between age at first childbirth and log-transformed triglyceride levels with scatter plots in total (A) and postmenopausal (B) women |
