# Supplementary material for: Age at first childbirth and the risk of hypertriglyceridemia among Korean women
Source: Epidemiol Health. 2022 Dec 29;45:e2023010. doi: 10.4178/epih.e2023010 (PMC10106550; doi:10.4178/epih.e2023010)
Supplement: Supplementary Material 2 — Cross-sectional association of age at first childbirth with prevalence of hypertriglyceridemia at baseline in total and postmenopausal women who had never used lipid-lowering medications [file epih-45-e2023010-Supplementary-Table-1.docx]

| Supplementary Material 2. Cross-sectional association of age at first childbirth with prevalence of hypertriglyceridemia at baseline in total and postmenopausal women who had never used lipid-lowering medications | | | | | | |
| --- | --- | --- | --- | --- | --- | --- |
| Age at first childbirth | No. of women | No (%) | Odds ratio (95% CI) for Hypertriglyceridemia | | | |
|  |  | of cases* | Unadjusted | Model 1 | Model 2 | Model 3 |
| Total women | |  |  |  |  |  |
| Continuous, | 15637 | 5012 (32.0) | 1.07 (1.06-1.08) | 1.02 (1.01-1.03) | 1.02 (1.01-1.03) | 1.01 (1.00-1.03) |
| per 1 year early |  |  |  |  |  |  |
| Category |  |  |  |  |  |  |
| <20 | 1111 | 459 (41.3) | 1.85 (1.62-2.12) | 1.21 (1.05-1.40) | 1.22 (1.06-1.41) | 1.20 (1.01-1.42) |
| 20-24 | 8665 | 2963 (34.2) | 1.37 (1.27-1.48) | 1.08 (1.00-1.18) | 1.09 (1.00-1.18) | 1.04 (0.94-1.15) |
| 25-29 | 5079 | 1398 (27.5) | 1.00 (ref) | 1.00 (ref) | 1.00 (ref) | 1.00 (ref) |
| ≥30 | 782 | 192 (24.6) | 0.86 (0.72-1.02) | 0.91 (0.76-1.09) | 0.92 (0.76-1.10) | 1.00 (0.79-1.26) |
| *p* for trend |  |  | <0.001 | 0.001 | 0.001 | 0.077 |
| Postmenopausal women | | | | | | |
| Continuous, | 11951 | 4217 (35.3) | 1.05 (1.04-1.07) | 1.02 (1.01-1.04) | 1.03 (1.01-1.04) | 1.01 (1.00-1.03) |
| per 1 year early |  |  |  |  |  |  |
| Category |  |  |  |  |  |  |
| <20 | 993 | 428 (43.1) | 1.68 (1.45-1.94) | 1.28 (1.09-1.49) | 1.29 (1.10-1.51) | 1.20 (1.02-1.42) |
| 20-24 | 7115 | 2602 (36.6) | 1.28 (1.17-1.39) | 1.12 (1.02-1.22) | 1.12 (1.02-1.23) | 1.04 (0.94-1.16) |
| 25-29 | 3389 | 1054 (31.1) | 1.00 (ref) | 1.00 (ref) | 1.00 (ref) | 1.00 (ref) |
| ≥30 | 454 | 133 (29.3) | 0.92 (0.74-1.14) | 0.91 (0.73-1.14) | 0.93 (0.75-1.16) | 0.99 (0.79-1.25) |
| *p* for trend |  |  | <0.001 | <0.001 | <0.001 | 0.070 |
| CI, confidence interval | | | | | | |
| *Cases for hypertriglyceridemia | | | | | | |
| Model 1: adjusted for age, study site, body mass index, menopausal status (only for total), blood pressure, and diabetes | | | | | | |
| Model 2: adjusted for age, study site, body mass index, menopausal status (only for total), blood pressure, diabetes, alcohol consumption, carbohydrate intake, income, marital status, and education | | | | | | |
| Model 3: adjusted for age, study site, body mass index, menopausal status (only for total), blood pressure, diabetes, alcohol consumption, carbohydrate intake, income, marital status, education, pairty, usage of oral contraceptives, and hormone replacement status | | | | | | |
|  |  |  |  |  |  |  |
