# Supplementary material for: Age at first childbirth and the risk of hypertriglyceridemia among Korean women
Source: Epidemiol Health. 2022 Dec 29;45:e2023010. doi: 10.4178/epih.e2023010 (PMC10106550; doi:10.4178/epih.e2023010)
Supplement: Supplementary Material 3 — Comparison of baseline characteristics between respondents and non-respondents to follow-up examinations [file epih-45-e2023010-Supplementary-Table-2.docx]

| Supplementary Material 3. Comparison of baseline characteristics between respondents and non-respondents to follow-up examinations | | | | | | | |
| --- | --- | --- | --- | --- | --- | --- | --- |
| Variables | Respondents to f/u | | | Non-respondents to f/u | | | p-value |
|  | (N=10,096) | | | (N=7,043) | | |  |
| Age, yrs | 58.3 | ± | 9.4 | 57.7 | ± | 9.5 | <0.001 |
| Age at first childbirth, yrs | 23.7 | ± | 3.4 | 23.9 | ± | 3.4 | <0.001 |
| Parity |  |  |  |  |  |  |  |
| 1-2 | 3454 | (33.7) | | 2162 | (30.4) | | <0.001 |
| 3-4 | 4276 | (41.7) | | 3090 | (43.4) | |  |
| ≥5 | 2320 | (22.6) | | 1740 | (24.4) | |  |
| Menarche age, yrs | 16.6 | ± | 2.9 | 17 | ± | 2.8 | <0.001 |
| Oral contraceptives | 2766 | (26.9) | | 2003 | (28.1) | | 0.214 |
| Hormone replacement therapy | 1137 | (15.0) | | 626 | (12.5) | | <0.001 |
| Postmenopausal status | 8002 | (77.7) | | 5470 | (76.3) | | 0.027 |
| Living with spouse | 8111 | (79.0) | | 5411 | (75.9) | | <0.001 |
| Low education level |  |  |  |  |  |  |  |
| (≤ Elementary school) | 6682 | (64.9) | | 4869 | (67.9) | | <0.001 |
| Low household income |  |  |  |  |  |  |  |
| (< 1,000 USD/month) | 5866 | (57.0) | | 4499 | (62.7) | | <0.001 |
| Occupation |  |  |  |  |  |  |  |
| White collar | 437 | (4.2) | | 236 | (3.3) | | <0.001 |
| Pink collar | 1187 | (11.5) | | 786 | (11.0) | |  |
| Blue collar | 5071 | (49.2) | | 3424 | (47.7) | |  |
| Others | 3606 | (35.0) | | 2728 | (38.0) | |  |
| Body mass index, kg/m² | 24.6 | ± | 3.2 | 24.5 | ± | 3.3 | 0.006 |
| Waist circumference, cm | 83.4 | ± | 9.0 | 82.4 | ± | 8.7 | <0.001 |
| Systolic BP, mmHg | 124 | ± | 17.9 | 127.3 | ± | 18.6 | <0.001 |
| Diastolic BP, mmHg | 77.7 | ± | 10.9 | 79.1 | ± | 11.4 | <0.001 |
| Fasting glucose, mg/dL | 92 | [87, 100] | | 91 | [85, 99] | | 0.026 |
| Fasting insulin, uIU/mL | 7.6 | [6.0, 9.9] | | 7.5 | [6.1, 9.7] | | 0.041 |
| Total cholesterol, mg/dL | 202.9 | ± | 37.1 | 205.3 | ± | 37.9 | <0.001 |
| HDL cholesterol, mg/dL | 46 | ± | 10.6 | 46.8 | ± | 11.1 | <0.001 |
| Triglycerides, mg/dL | 120 | [87, 170] | | 119 | [86, 171] | | 0.049 |
| Hypertriglyceridemia | 3354 | (32.6) | | 2371 | (33.1) | | 0.497 |
| Dyslipidemia | 4884 | (49.4) | | 3420 | (49.9) | | 0.471 |
| Diabetes | 1125 | (10.9) | | 810 | (11.3) | | 0.444 |
| Hypertension | 1676 | (16.3) | | 541 | (7.5) | | <0.001 |
| Physical activity | 3256 | (31.7) | | 2176 | (30.4) | | 0.082 |
| Current smokers | 240 | (2.3) | | 185 | (2.6) | | 0.295 |
| Current drinkers | 2809 | (27.3) | | 2014 | (28.1) | | 0.242 |
| Energy intakes, kcal/day | 1575.8 | ± | 549.4 | 1596.8 | ± | 578.0 | 0.016 |
| Values are shown as mean ± SD, median [IQR], or number (%). | | | | | | | |
| BP, blood pressure; HDL, high-density lipoprotein. | | | | | | | |
