# Supplementary material for: Age at first childbirth and the risk of hypertriglyceridemia among Korean women
Source: Epidemiol Health. 2022 Dec 29;45:e2023010. doi: 10.4178/epih.e2023010 (PMC10106550; doi:10.4178/epih.e2023010)
Supplement: Supplementary Material 4 — Longitudinal association between age at first childbirth at the baseline visit and incidence of hypertriglyceridemia at a follow-up visit among participants who underwent at least three follow-up examinations [file epih-45-e2023010-Supplementary-Table-3.docx]

| Supplementary Material 4. Longitudinal association between age at first childbirth at the baseline visit and incidence of hypertriglyceridemia at a follow-up visit among participants who underwent at least three follow-up examinations | | | | | | | |
| --- | --- | --- | --- | --- | --- | --- | --- |
| **Age at first childbirth** | **Numbers at risk** | **Events*** | **Person-years** | **Incidence rate** | **Hazard ratio (95% CI) for hypertriglyceridemia** | | |
|  |  |  |  | **(/100pys)** | **Unadjusted** | **Model 1** | **Model 2** |
| **Total women** |  |  |  |  |  |  |  |
| Continuous, per 1 year early | 1552 | 575 | 10991.5 | 5.2 | 1.03 (1.00-1.05) | 1.02 (0.99-1.05) | 1.042 (1.003-1.081) |
| Category |  |  |  |  |  |  |  |
| <20 | 91 | 43 | 628.9 | 6.8 | 1.41 (1.01-1.96) | 1.27 (0.89-1.81) | 1.69 (1.11-2.58) |
| 20-24 | 809 | 315 | 5812.7 | 5.4 | 1.14 (0.95-1.36) | 1.08 (0.89-1.32) | 1.35 (1.05-1.74) |
| 25-29 | 544 | 187 | 3977.5 | 4.7 | 1.00 (ref) | 1.00 (ref) | 1.00 (ref) |
| ≥30 | 78 | 30 | 572.4 | 5.2 | 1.09 (0.74-1.60) | 1.04 (0.71-1.54) | 0.92 (0.541-1.57) |
| *p* for trend |  |  |  |  | 0.075 | 0.2855 | 0.005 |
| CI, confidence interval | | | | | | | |
| *Hypertriglyceridemia events | | | | | | | |
| Model 2: adjusted for age, study site, body mass index, menopausal status (only for total), blood pressure, diabetes, alcohol consumption, carbohydrate intake, income, marital status, and education. | | | | | | | |
| Model 2: adjusted for age, study site, body mass index, menopausal status (only for total), blood pressure, diabetes, alcohol consumption, carbohydrate intake, income, marital status, education, parity, usage of oral contraceptives, and hormone replacement status | | | | | | | |
